# Anomalous altered expressions of downstream gene-targets in TP53-miRNA pathways in head and neck cancer

**DOI:** 10.1038/srep06280

**Published:** 2014-09-04

**Authors:** Sanga Mitra, Nupur Mukherjee, Smarajit Das, Pijush Das, Chinmay Kumar Panda, Jayprokas Chakrabarti

**Affiliations:** 1 Computational Biology Group, Indian Association for the Cultivation of Science, Kolkata, India; 2 Department of Oncogene Regulation, Chittaranjan National Cancer Research Institute, Kolkata, India; 3 Department of Medical Biochemistry and Cell Biology, Institute of Biomedicine, University of Gothenburg, Sweden; 4 Cancer Biology & Inflammatory Disorder Division, Indian Institute of Chemical Biology, Kolkata, India; 5 Gyanxet, BF 286 Salt Lake, Kolkata, India

## Abstract

The prevalence of head and neck squamous cell carcinoma, HNSCC, continues to grow. Change in the expression of TP53 in HNSCC affects its downstream miRNAs and their gene targets, anomalously altering the expressions of the five genes, MEIS1, AGTR1, DTL, TYMS and BAK1. These expression alterations follow the repression of TP53 that upregulates miRNA-107, miRNA- 215, miRNA-34 b/c and miRNA-125b, but downregulates miRNA-155. The above five so far unreported genes are the targets of these miRNAs. Meta-analyses of microarray and RNA-Seq data followed by qRT-PCR validation unravel these new ones in HNSCC. The regulatory roles of TP53 on miRNA-155 and miRNA-125b differentiate the expressions of AGTR1 and BAK1in HNSCC vis-à-vis other carcinogenesis. Expression changes alter cell cycle regulation, angiogenic and blood cell formation, and apoptotic modes in affliction. Pathway analyses establish the resulting systems-level functional and mechanistic insights into the etiology of HNSCC.

In recent years, the onset and prevalence head and neck cancer (HNSCC) in Asia have been fairly well documented. In India, for instance, about 33% of all cancer cases per year are HNSCC, and the National Cancer Registry Programme of the Indian Council of Medical Research in 2010 estimated that HNSCC cases are going to increase significantly by 2020^1^. Genetic and miRNA expression alterations are implicated in HNSCC^2^,^3^. miRNAs target several transcripts and affect their expressions, and are established as prognostic markers in HNSCC^2^. Thus, misregulation of miRNA expression may lead to various disorders and diseases including cancer, and hence miRNA expression regulation was in focus^4^. Transcription factors, TFs, directly regulate genes/miRNAs, or indirectly regulate other genes via miRNA or genes^5^,^6^. The TF TP53 gene, located on chromosome 17p13.1, is involved in a wide variety of cancers including HNSCC^7^. TP53 is implicated in several pathways, such as cell cycle, Wnt signaling, MAPK signaling, transcriptional misregulation in cancer^8^,^9^. TP53, once activated, leads to apoptosis and growth arrest (either cell cycle arrest or senescence)^10^. Input from several experimental data including NGS has made clear that TP53 mutation is common in HNSCC^11^,^12^. Moreover, TP53 mutation gains in importance for risk factors of alcohol and tobacco^13^. With increasing TP53 mutation, progression from premalignancy to malignancy is accelerated^14^. Manifold functionalities and implications of TP53 make it the choice for analysis.

TP53, being downregulated in HNSCC, fails to regulate a number of miRNAs, resulting in altered expressions downstream. How the alterations of miRNAs under transcriptional control of TP53 changed the fate of their downstream targets were the goals of this study^15^,^16^. The investigation took into consideration only those miRNAs that were relevant to HNSCC, the ones that were TP53-influenced. These miRNAs were dubbed TP53-miRNAs. TP53-miRNAs, such as miRNA-107, miRNA-215, miRNA-34b/c and miRNA-125b, were downregulated in HNSCC, but, noticeably, miRNA-155 remained upregulated. The interesting sequel to TP53 mediated miRNA regulation was the perturbed expressions of their downstream gene targets. Statistical investigations using microarray datasets and RNA-seq datasets lent a measure of credence to the results, and some of the altered gene-expressions were new in HNSCC pathways. The expression status of the new candidate genes, MEIS1, LDOC1, AGTR1, BAK1, TYMS and DTL, were validated through qRT-PCR. Figure 1 gives an overall view of the workflow. It is proposed that two tumor suppressor genes, MEIS1and AGTR1, and one oncogene, BAK1, are involved in HNSCC.

## Results

### TP53-miRNA Network

**Figure 2:**
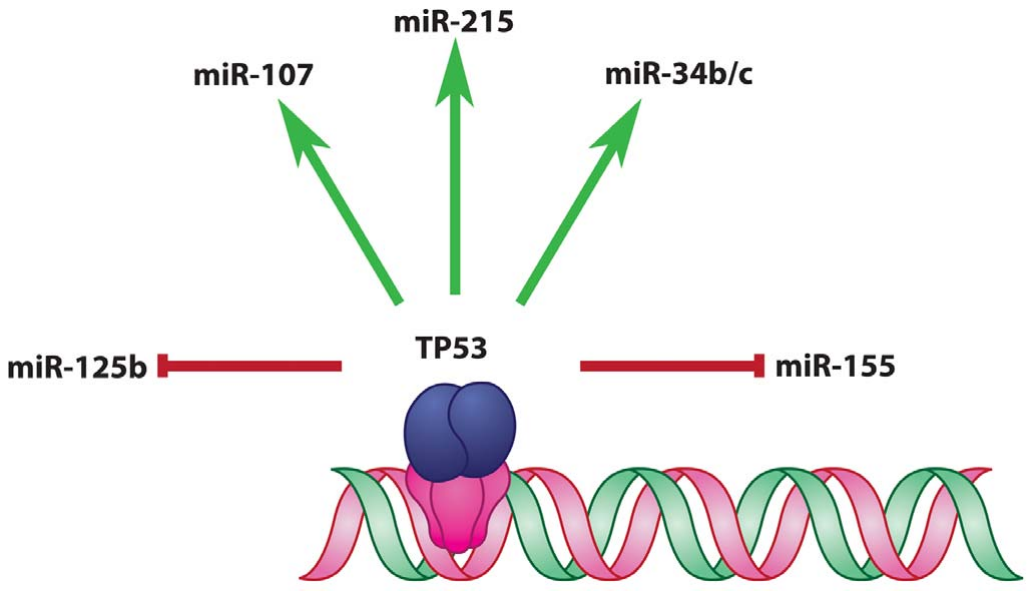
TP53-miRNA Network: Transcription factor, TP53, controlling HNSCC relevant miRNAs, in normal condition, is depicted in this network.

To visualize the transcriptional regulation of miRNAs by TP53, the TP53 controlled miRNAs, TP53-miRNAs, were extracted from TransmiR database^17^. These miRNAs were mapped to HNOCDB^18^, and checked further with published literature^19^,^20^, only the ones relevant to HNSCC were retained for the study. As evident from TransmiR, in normal condition, the TP53-miRNA network had three activation links and two repression links, TP53 upregulated miRNA-107, miRNA-215 and miRNA-34 b/c, but downregulated miRNA-125b and miRNA-155 (Figure 2). In concert with TP53, these miRNAs were involved in cell regulation^16^. TP53 generally binds to the decamer motif (‘RRRCWWGYYY’, where ‘R’ represents purine and ‘W’ is either ‘A’ or ‘T’) present in tandem, with a spacer of 0–13 nucleotides in between, the motif being present upstream of the concerned gene/miRNA regulated by TP53^21^.

**Figure 3:**
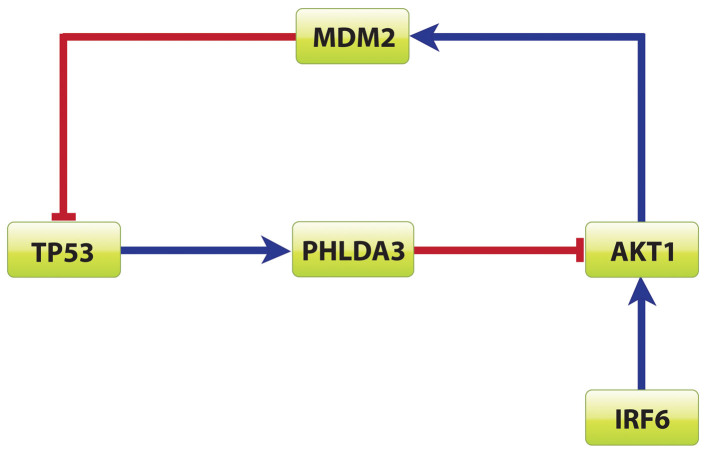
Negative feedback loop involving TP53 and AKT1: In presence of AKT1, TP53 is silenced by MDM2, whereas TP53 in its presence represses AKT1 via PHLDA3. AKT1 in turn is upregulated by IRF6 in HNSCC.

In most cancers, TP53 is mutated, indicating the important role played by TP53 as a tumor suppressor^22^,^23^. TP53 was reported the most frequently mutated gene in HNSCC by Agarwal et.al^11^. The change of TP53 status had effects on its downstream miRNA. The expression status of the following five miRNAs in HNSCC, from HNOCDB^22^ and PhenomiR2^24^, were: miRNA-107, miRNA-215, miRNA-34b/c and miRNA-125b were downregulated, but miRNA-155 was augmented in the absence of TP53. Notice the unexpected result for miRNA-125b: it was anticipated to be upregulated in TP53's dearth. On further investigation, it was found the AKT1 binding motif was present upstream of miRNA-125b. AKT1, activated by IRF6 (LPS), differentially regulated several miRNAs. AKT1 and TP53 formed a negative feed-forward loop^25^ (Figure 3). In the absence of TP53, AKT1 repressed miRNA-125b and hence was downregulated^26^. Both IRF6 and AKT1 were already reported to be upregulated in HNSCC^27^. In cancer biology, different kinds of networks are formed whose dynamic properties are interpreted based on several models^28^. Over here, the negative feedback motif was constructed based on data-mining and microarray data^29^. In this circuit, IRF6 positively induced AKT1 that upregulated MDM2, which in turn repressed TP53 in HNSCC. Taking a cue from previously published data^25^,^26^,^27^,^28^, IRF6 was the input node that first sent the signal to the circuit, the transient molecules acted accordingly, and finally TP53 was the repressed output in this negative feedback loop.

### miRNA-Target Selection

To find out the downstream effects of TP53-miRNA interaction, the validated targets of these five miRNAs from miRecords were selected^30^. A total of 90 targets were found. The goal was to determine which of these targets were altered in HNSCC. These alterations were investigated first from analysis of microarray data followed by RNA sequencing data.

### Expression Data Analysis

#### Microarray Data

**Figure 4:**
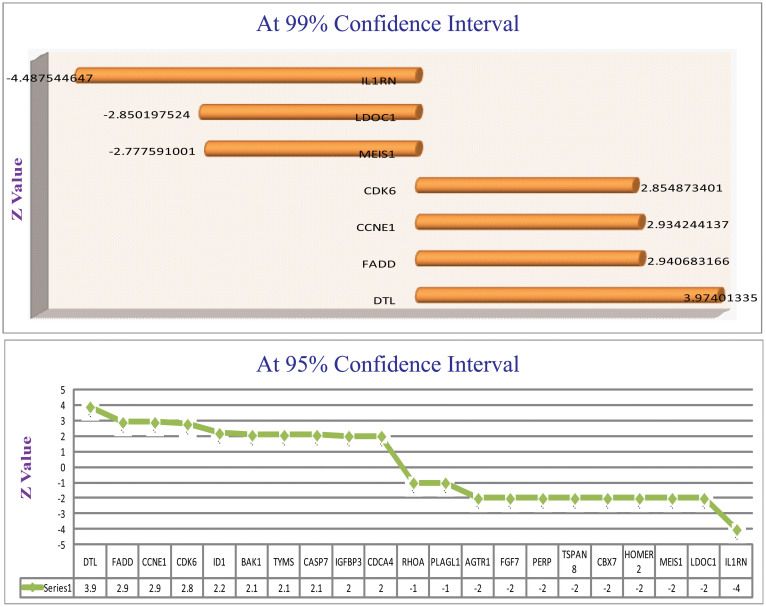
Microarray Meta analysis Results: z-values of the targets filtered at 95% and 99% confidence interval are pictorially represented. The novel genes are filtered from this potential gene list.

HNSCC microarray data sets were downloaded from Oncomine version 4.4.4.4[https://www.oncomine.org/resource/login.html]. Six datasets were taken under consideration. From these 90 targets, to predict the most potential candidates, i.e. those actually altered in HNSCC, the expression values (t-values in this case) were noted from individual datasets. At 95% significant level, the targets lining on either side of the critical values were selected^31^. Based on these values of the targets, collected from all the datasets, Z statistics was applied and support from the z-value was used to rank the considered 90 genes^32^. Targets were filtered at both 95% confidence interval and 99% confidence interval of z-value (Figure 4). The targets qualifying 95% confidence interval were chosen for further study. Based on microarray data analysis, the strong oncogenic and tumor suppressive hubs were located. These selected targets were compared with HNSCC gene list from HNOCDB. From this comparison, it transpired that not all the oncogenes and tumor suppressor genes were known. Six gene-targets, namely, DTL, TYMS, BAK1, AGTR1, MEIS1 and LDOC1 were new.

**Figure 5:**
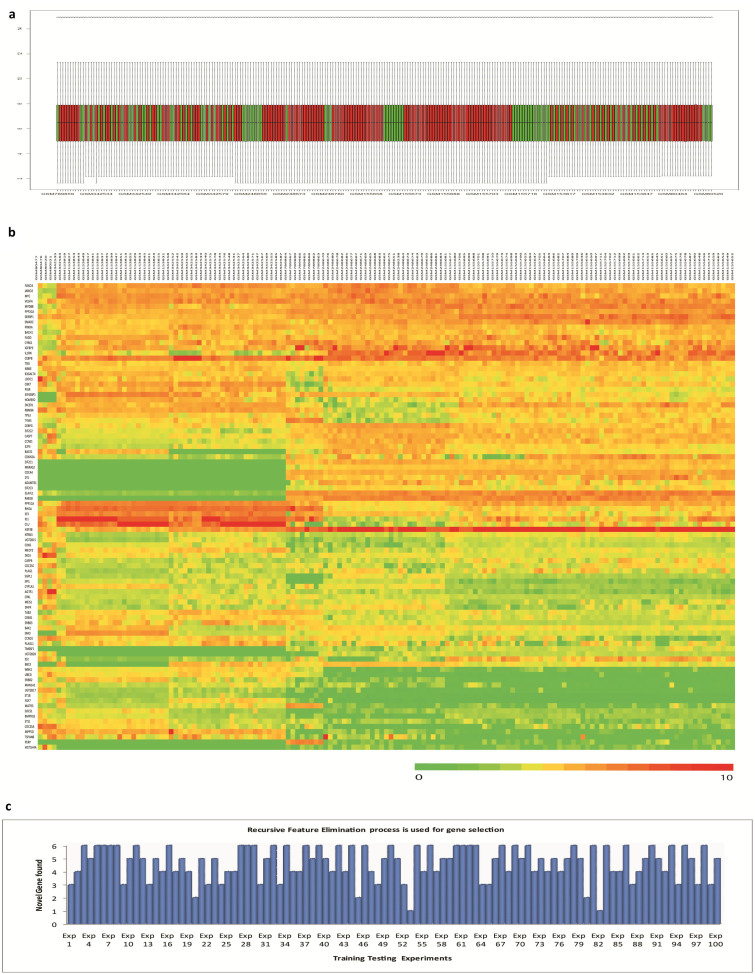
Robust Gene-Set Identification by Re-sampling Method: (a). Normalized data are represented by a box plot. Here in this graph, the red color represents tumor sample and the green color represents normal sample. (b). The normalized intensity values of our selected 90 target genes, taken from GEO datasets, are represented by a heat map. (c). The abundance of the novel genes found after each re-sampling operation is represented graphically.

To remove any sampling bias, the data was reanalyzed^33^. The raw values of the used Oncomine datasets were downloaded from GEO database [http://www.ncbi.nlm.nih.gov/geo/]. Since all the datasets were retrieved from affymetrix platform, thus it was not required to produce platform independent data set for this analysis. The data matrix was normalized using the limma bioconductor package [http://www.bioconductor.org/]. The expression intensities were normalized so that the intensities, or the log-ratios, had similar distributions across a set of arrays. The normalized box plot is in figure 5a. After normalization, k-means clustering was used to find out the outliers. After removing the outlier, the differential expression operation was performed as the data set had unequal tumor and normal sample pool. The normalized intensity values were then used to produce the heat map, Figure 5b. Now, to produce a robust gene set, the recursive feature elimination procedure was implemented. 1/3 of the normalized data was randomly selected as the training set to predict 2/3 of the sample. For class prediction between Tumor and Normal, SVM method was used. The gene list was collected from training dataset by using recursive feature elimination procedure, and the test set was predicted. The number of feature selected each time was 20 from the test gene list, and each time tumor class and normal class were distinguished at more than 80% accuracy rate. This training-testing procedure was repeated a 100 times. The abundance of the novel genes inside the feature element set was 78%. In figure 5c the graphical representation of the appearance of the novel gene list is given.

#### RNA-Sequencing Data

From The Cancer Genome Atlas (TCGA) [http://cancergenome.nih.gov/], HNSCC data of 22 treated samples and 4 untreated samples were downloaded. The previously explored 90 targets were again used for the NGS study. The goal was to determine through NGS analysis which of these targets were altered in HNSCC. Based on the read count of the samples, class prediction test, a supervised learning method was applied^34^. The prediction paradigm served as a good framework for comparing different prediction methods and as a result gave non-spurious findings. By analyzing this data with these statistics, 6 most potential biomarkers (genes) to classify 26 samples (22 cases, 4 control), with 80–95% accuracy level, were recorded. These six genes resulted in as a good classifiers of the treated and untreated samples (Table 1) and concerned a future clinical outcome. Among those 6 genes, 4 were considerably down-regulated, and rest 2 significantly up-regulated. Out of these six candidates, DTL and MEIS1 matched the selected list from microarray data analysis. Of the remaining four, two genes, MATR3 and EIF2C2, were not related to HNSCC to date. Before considering these as novel candidates, negative binomial test^35^ on these 26 samples was performed, and calculated the fold change, t-value, p-value and q-value. Since q-value^36^ was considered as good director of the expression status, based on this q-value (<0.05) it was observed that MATR3 and EIF2C2 did not qualify as good candidate genes, but DTL and MEIS1 still held strongly. DTL in this dataset was also found to be overexpressed, while MEIS1was repressed.

Validated through NGS, these two, and in addition the previous four from microarray data analysis, were our new candidate genes for HNSCC. However, before final confirmation, validation of these genes through qRT-PCR was undertaken.

#### Experimental Validation

**Figure 6:**
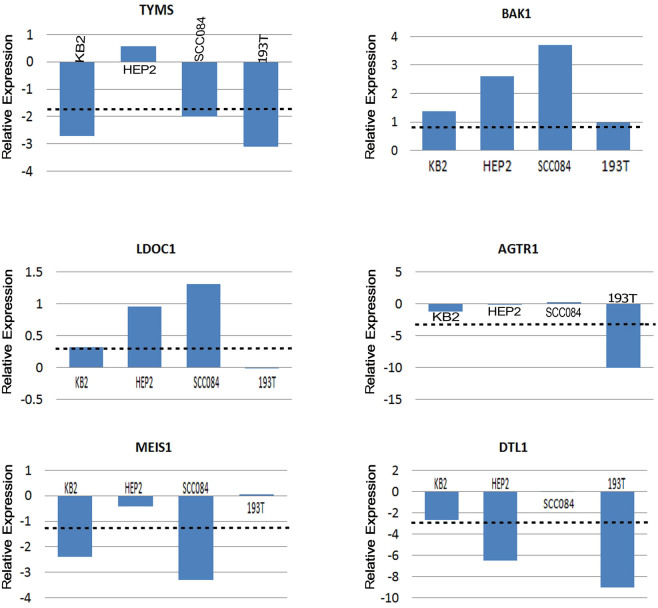
Graph derived from qRT-PCR Analysis: Quantitative RT-PCR analysis showing reduced/increased expression of TYMS, BAK1, LDOC1, AGTR1, MEIS1 and DTL1 samples in KB2, HEP2, SCC084 cell line and one HNSCC primary tumor sample. Bars represent the gene expression normalized to b2-microglobulin and relative to a pool of normal oral tissues. The line illustrates the mean reduction level of respective genes. X-axis indicates samples.

mRNA expression of the candidate genes MEIS1, LDOC1, AGTR1, BAK1, TYMS and DTL were analyzed in 3 cell lines (Hep2, KB and SCC 084), 1 primary HNSCC tumor and 3 normal head and neck tissue samples. Based on this in-vivo analysis, MEIS1 and AGTR1 (repressed) and BAK1 (overexpressed) showed the desired expression status as obtained through in-silico analysis, but LDOC1 had insignificant change. Interestingly, TYMS and DTL showed stark reversal to what was obtained from microarray and RNA-seq data analysis (Figure 6). This discrepancy may have arisen due to ethnic variations in populations or differences in methods of detection between the data types used in array and NGS data sets and the sample used for in-house validation. Further functional validation is required to account for this inconsistency.

## Discussion

Amalgamation of in-silico analysis (network analysis and expression data analysis) and experimental validation (qRT-PCR) revealed alterations in the expression levels of five new genes in HNSCC. Exploration of TP53-miRNA-Target pathway helped discover these novel oncogenes and tumor-suppressor genes of HNSCC. Considering the functionalities of these genes, it was found that cell cycle regulation, angiogenic, blood cell formation and apoptotic pathways were encompassed by the expression changes of the newly found HNSCC-relevant genes. The functional details were scanned using Gene Ontology tool [http://amigo.geneontology.org/amigo] and depicted in Table 2 along with their choromosomal locations.

Highly expressive miRNA-155 led to silencing of AGTR1 and MEIS1. AGTR1 was involved in blood pressure control, whereas MEIS1 regulated megakaryocytic lineage. Angiotensin and its receptor, AGTR (Angiotensin receptor) were known as main effectors of renin-angiotensin system, a hormonal signaling mechanism implicated in the regulation of blood pressure and cardiovascular homeostasis. Angiotensin II type 1 receptor (AGTR1), one of the receptors of angiotensin II (AGTII). AGTII, via AGTR1, exerts stimulatory actions on angiogenesis, cell growth and cell proliferation in tissues. In several instances AGTR1 was reported to be overexpressed in breast cancer^37^,^38^. However, remarkably in HNSCC, AGTR1 was downregulated. It appeared that AGTR1 was under repressive control of miRNA-155, an oncomiR for HNSCC. Experimental validation through qRT-PCR on HNSCC cell lines KB, SCC084, SCC131 and tumor sample 193T established the repressed nature of AGTR1 in HNSCC. It was considered that promoter of AGTR1 may have been hypermethylated; as a result was silenced. Methylation mediated gene silencing often led to tumorogenesis. In ovarian cancer promoter of AGTR1, it was reported to be hypermethylated, whereas no methylation mark for normal ovarian epithelium^39^. In a single report, the degree of AGTR1 methylation was shown to be significantly high for patients with oral preneoplastic lesions^40^. These two previous instances, and the computational and experimental effort detailed here, establish AGTR1 as a potential tumor suppressor gene in HNSCC.

Similar to the fate of AGTR1, MEIS1 also remained silenced in HNSCC. The MEIS1 homeobox gene is known to be involved in several hematological malignancies and solid tumors. Recent evidence suggested that expression of the MEIS1 transcript was altered in colorectal cancer. Both the homeodomain-less isoforms of MEIS1, i.e., MEIS1D transcript and protein (27KD and 32KD), were downregulated in primary colorectal cancer samples compared to matched normal mucosa, indicating that MEIS1D was a biomarker of colorectal tumorigenesis^41^. The decreased expression of MEIS1 was also observed for prostate poor-prognosis tumors, suggesting that MEIS1 might act as a tumor suppressor in human prostate cancer^42^. CpG island, and promoter hypermethylation, downregulated MEIS1 in lung squamous cell carcinoma^43^, and adenoid cystic carcinoma^44^ respectively. In contrast to all these studies, MEIS1, in concert with PBX and HOXA9, acted as oncogenes in leukemia, neuroblastoma and ovarian cancer^45^,^46^,^47^. In the present study, evidence was found that MEIS1 gene expression was downregulated in HNSCC both through in silico and in vitro analysis. Previous study of EIS1 fate in other cancers indicated that either MEIS1 promoter methylation, or truncated form of MEIS1, was associated with decreased MEIS1 gene expression in HNSCC. Both miRNA-155 targets, i.e. MEIS1 and AGTR1, were silenced via miRNA-155 upregulation, and seemed to have been influenced by promoter methylation.

miRNA-215 was known to contribute to cell cycle arrest and cell detachment from firm support. This showed that targets of miRNA-215, DTL and TYMS, were linked to cell cycle regulation. DTL helped to establish genomic stability through two distinct mechanisms. First, DTL, being an essential component of the CUL4-DDB1 complex, regulated the level of CDT1. Second, it was required for early G2/M checkpoint^48^. DTL played an essential role in cell proliferation, cell cycle arrest and metastatic potential in heptacellular carcinoma, breast cancer, gastric cancer and rhabdomysarcoma. It has been observed that miRNA-215, via DTL, was able to suppress cell growth in osteosarcoma and colon cancer cells. Thus, DTL overexression in cancer was correlated with bigger tumor size, particularly where p53 was mutated^48^. In stark reversal to this, it was found in our in-vitro analyses that DTL was many-fold downregulated in HNSCC cell lines and in tumor samples. The probable effect of loss of DTL from HNSCC cell line must have been the rereplication of DNA, which was indicative of a failure to inhibit CDT1 during S phase. DTL was required for both CDT1 degradation and inhibition of replication during the normal cell cycle of human cells. DNA damage dictated DTL to downregulate CDT1, failure of which led to genomic instability and cancer. Depletion of DTL caused rereplication and appearance of multipolar spindles, spindle formation characteristics of cancer cells^49^. This was surmised to be the probable reason for connection of DTL with HNSCC. Moreover, DTL protein was localized at specific sites at the end of chromosome, and had a role in telomere protection pathway. The molecular mechanism by which DTL participated in telomere maintenance is unknown. Loss of DTL abrogated its role of telomere maintenance and led to telomere association, a cytogenetic phenomenon where chromosome ends fused to dicentric, multicentric and ring chromosomes. Such conditions of chromosomes also led to carcinogenesis^50^. Therefore, it seemed that DTL followed a significantly different course in HNSCC.

Another miRNA-215 controlled effector, i.e. Thymidylate synthetase, a key enzyme of folate metabolism, catalyzed conversion of deoxyuridine monophosphate (dUMP) to deoxythymidine monophosphate (dTMP) leading to production of thymidine, an important component of DNA required for its repair and synthesis. TYMS, also called TS, mainly aided in rapid cell division and growth. TS gene was mainly expressed in S-phase and G2 phase and its expression was elevated in rapidly proliferating cells compared to non-dividing cells. Folate metabolism being essential to both cancerous and normal cells, depletion of TYMS and its resultant diminution of DNA synthesis and DNA methylation must be toxic to both malignant cells and normal cells. Polymorphism of TYMS gene promoter enhancer region (TSER) at 5′UTR, consisting of VNTR, dictated the TS mRNA expression level and translation efficiency. Presence of VNTRs generated several genotypes of TYMS genes, most common having double (2R) and triple tandem repeats (3R), while other may have four and nine repeats. Among the homozygous 2R/2R, heterozygous 2R/3R and homozygous 3R/3R, the later allelic variety produced the greatest measure of TYMS protein product^51^. TYMS, as reported until date, showed high level of expression in almost all cancers, starting from breast, colorectal, renal, gastric and several others^52^,^53^. Overexpression of TYMS mRNA and protein resisted chemotherapy in patients suffering from rectal, gastric and HNSCC^54^. Interestingly, here evidence was produced that TYMS gene was downregulated. This left us quizzing what could be the possible reason for loss of TYMS function in malignant condition. Loss of allelic band 18p11.32 with TYMS embedded in it was previously reported only in case of renal cell carcinoma^55^. TYMS enzyme responsible for folate acid metabolism led to cell proliferation, hence thought to sustain malignancy. Paradoxically, inhibition of folic acid metabolism has been used as a mechanism for successful elimination of malignant cells, but insufficient folic acid levels in cells have been associated with DNA damage and altered DNA methylation conditions associated with malignant transformation. Interference, or prevention of folic acid metabolism, was then potentially both a cause of malignancy and a means to induce apoptosis or necrosis in malignant cells. The complexity of the folic acid metabolic cycle and its use in construction and modification of fundamental biomolecules of the cell allowed this paradox to exist^56^. For both the miRNA-215 controlled genes, DTL and TYMS, incongruities of expression levels between the in-silico derived conclusion and in-vitro validation were observed. Hence, further functional test for both miRNA and genes has to be performed in future.

Lack of expression of miRNA-125b boosted the level of BAK1, thus controlling the apoptotic pathway. BAK1, a homologue of BCL-2, is a potential inducer, either pro or anti of apoptosis. BAK1 was seen to be overexpressed in patients affected with autoimmune disease such as multiple sclerosis, MS. MS predisposed patient to high risk of cancer. Single nucleotide polymorphism of BAK1 made patients susceptible to autoimmune lesions. Similarly SNP, in or near BAK1, prompted the occurrence of testicular germ cell tumor. Generally, BAK1 was downregulated in many cancers under the influence of miRNA. miRNA-150 downregulated BAK1 in non-small-cell lung cancer^57^ and in the same way miRNA-125b downregulated BAK1 in cervical cancer, breast cancer and prostate cancer^58^,^59^,^60^. In a remarkable contrast, miRNA-125b was found to be downregulated in HNSCC, and as a result, its target BAK1 shot up in its expression in HNSCC.

These observations added to details of genetic integrity of HNSCC. With increasing study of network biology, the importance of integrating transcription factor, miRNA and their target genes in a single network became important. It was well established here that TF and miRNA acted cooperatively to regulate target genes. Thus TP53 along with its downstream cohorts formed a key hub in HNSCC network. Though past studies have identified several genes associated with HNSCC, the genes discussed here were new. Deeper understandings of the implications of altered expressions of MEIS1, AGTR1, BAK1, DTL and TYMS, call for detailed clinical studies for further insights into HNSCC pathogenesis.

## Methods

To build up the TP53-miRNA network, manually curated TP53 controlled miRNAs from the TF-miRNA regulation database, TransmiR, were assembled. The HNSCC databases, HNOCDB and PhenomiR2, containing differentially regulated miRNA expression in diseases and other biological processes, were used for corroboration, and further extended this network by extracting validated miRNA-gene-targets from miRecord, a resource for animal miRNA-target interactions.

Next, for the expression data (microarray and NGS) analysis, Oncomine and TCGA were utilized. Six microarray datasets were downloaded from ONCOMINE 4.4.4.4, namely: Estilo Head-Neck, Hensen Head-Neck, Kuriakose Head-Neck, Pyeon Multi-cancer, Toruner Head-Neck and Ye Head-Neck. On these datasets, t-statistics and z statistics were applied to obtain potential HNSCC genes, and cross validated using resampling method. The raw datasets corresponding to the already used oncomine datasets were retrieved from GEO database by using the R bioconductor package termed GEOquery. The affymatrix array datasets with following NCBI GEO IDs: GSE3524, GSE6631, GSE6791, GSE9844, GSE13601 and GSE31853 were used. Total 255 samples were used. First, it was checked whether each array matrix was log2 transformed, if not, then all data matrix were appropriately changed. Then the intensity values of the selected 90 target genes were fetched from each data set corresponding to each gene name. All the intensity values from each dataset were then combined to produce a combined data matrix. The data matrix was then normalized by using limma bioconductor package. K-means clustering was used to remove the outliers and then differential expression operation was performed using lumi bioconductor package on the remaining datasets. Samples were randomized for every operation. Recursive feature elimination procedure was used to select significant genes. Finally, class prediction method was performed using SVM to classify normal and tumor samples. For recheck, and probable new input, RNA-sequencing data of 22 treated samples and 4 untreated samples of HNSCC from The Cancer Genome Atlas (TCGA) were used. The data belonged to Batch 83 of HNSCC Data Matrix downloaded from TCGA portal. Class prediction followed by negative binomial test, using BRB-Array tool and R-packages, were applied on NGS data. By merging microarray and NGS data 6 potential new genes were obtained, and were subsequently subjected to qRT-PCR confirmation.

mRNA expression of the candidate genes MEIS1, LDOC1, AGTR1, BAK1, TYMS and DTL were analyzed in 3 cell lines (Hep2, KB and SCC 084), 1 primary HNSCC tumor and 3 normal head and neck tissue samples. Total RNA was isolated using TRIzol reagent according to the manufacturer's protocol (Invitrogen, USA). Real-time quantification of the candidate genes was performed using a power SYBR-green assay (Applied Biosystems, USA) according to the standard protocols with β2-Microglobulin as control. The comparative threshold cycle (ddC_T_) method was employed to determine the relative level of gene expression (Livak and Schmittgen 2001).

(See Supplementary Materials for details of Microarray and NGS analysis, qRT-PCR method and sequences of primers in Table S1).

## Supplementary Material

Supplementary Information Supplementary Information

## Figures and Tables

**Figure 1:**
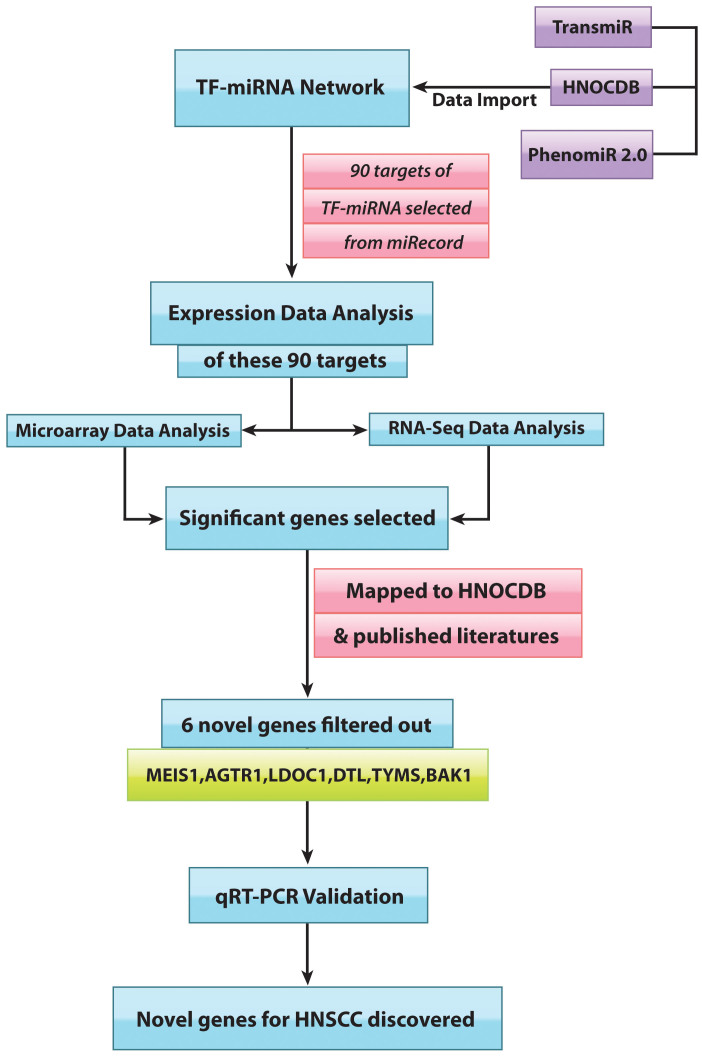
Flowchart of the study: It portrays the direction that was followed to identify the novel set of genes for HNSCC.

**Table 1:** Composition of classifier

Sorted by t –value; Class 1: *treated*; Class 2: *untreated*							
	Parametric p-value	t-value	% CV support	Geom mean of intensities in class 1	Geom mean of intensities in class 2	Fold-change	UniqueID
1	3.4e-06	−6	100	925.27	6910.09	0.13	MEIS1
2	5.4e-06	−5.811	100	16675.06	44388.62	0.38	ERBB2
3	0.0007684	−3.85	69	9828.1	26238.49	0.37	ERBB3
4	0.0008098	−3.829	46	24373.69	34259.58	0.71	MATR3
5	0.0007551	3.857	69	2800.48	694.82	4.03	DTL
6	4.48e-05	4.97	100	4086.13	1342.36	3.04	EIF2C2

**Sorted by Gene Weights**				
	Genes	Compound Covariate Predictor	Diagonal Linear Discriminant Analysis	Support Vector Machines
1	DTL	3.8573	2.186	0.0607
2	EIF2C2	4.9704	4.5451	0.623
3	ERBB2	−5.8106	−7.0623	−0.7272
4	ERBB3	−3.8504	−3.0919	−0.3101
5	MATR3	−3.8295	−8.8212	−0.0482
6	MEIS1	−5.9996	−3.6662	−0.7002

**Table 2:** Significant gene ontology terms related to five novel altered genes

GENE NAME		ENTREZ GENE ID	CHROMOSOMAL LOCATION	GENE ONTOLOGY FUNCTIONS	
MEIS1	Meis homeobox 1	4211	2p14	sequence-specific DNA binding transcription factor activity	GO:0003700
AGTR1	Angiotensin II receptor, type 1	185	3q24	angiotensin type I receptor activity	GO:0001596
				angiotensin type II receptor activity	GO:0004945
				angiotensin-activated signaling pathway	GO:0038166
				bradykinin receptor binding	GO:0031711
				calcium-mediated signaling	GO:0019722
				cell chemotaxis	GO:0060326
				G-protein coupled receptor signaling pathway	GO:0007186
				integral component of membrane	GO:0016021
				integral component of plasma membrane	GO:0005887
				kidney development	GO:0001822
				low-density lipoprotein particle remodeling	GO:0034374
				phospholipase C-activating angiotensin-activated signaling pathway	GO:0086097
				phospholipase C-activating G-protein coupled receptor signaling pathway	GO:0007200
				positive regulation of cellular protein metabolic process	GO:0032270
				positive regulation of cholesterol esterification	GO:0010873
				positive regulation of cytosolic calcium ion concentration	GO:0007204
				positive regulation of inflammatory response	GO:0050729
				positive regulation of macrophage derived foam cell differentiation	GO:0010744
				positive regulation of NAD(P)H oxidase activity	GO:0033864
				positive regulation of phospholipase A2 activity	GO:0032430
				positive regulation of reactive oxygen species metabolic process	GO:2000379
				protein heterodimerization activity	GO:0046982
				regulation of blood vessel size by -angiotensin	GO:0002034
				regulation of cell growth	GO:0001558
				regulation of cell proliferation	GO:0042127
				regulation of inflammatory response	GO:0050727
				regulation of renal sodium excretion	GO:0035813
				regulation of systemic arterial blood pressure by renin-angiotensin	GO:0003281
				regulation of vasoconstriction	GO:0019229
				regulation of vasodilation	GO:0042312
				renin-angiotensin regulation of aldosterone production	GO:0002018
				Rho protein signal transduction	GO:0007266
DTL	Denticleless homolog (Drosophila)	51514	1q32.3	cellular response to DNA damage stimulus	GO:0000674
				Cul4B-RING E3 ubiquitin ligase complex	GO:0031465
				DNA replication	GO:0006260
				G2 DNA damage checkpoint	GO:0031572
				protein monoubiquitination	GO:0006513
				protein polyubiquitination	GO:0000209
				regulation of cell cycle	GO:0051726
				response to UV	GO:0009411
				translesion synthesis	GO:0019985
				ubiquitin-dependent protein catabolic process	GO:0006511
				ubiquitin-protein transferase activity	GO:0004842
TYMS	Thymidylate synthetase	7298	18p11.32	aging	GO:0007568
				cartilage development	GO:0051216
				circadian rhythm	GO:0007623
				deoxyribonucleoside monophosphate biosynthetic process	GO:0009157
				developmental growth	GO:0048589
				DNA repair	GO:0006281
				DNA replication	GO:0006260
				dTMP biosynthetic process	GO:0006231
				dTTP biosynthetic process	GO:0006235
				dUMP metabolic process	GO:0046078
				folic acid binding	GO:0005542
				G1/S transition of mitotic cell cycle	GO:0000082
				immortalization of host cell by virus	GO:0019088
				intestinal epithelial cell maturation	GO:0060574
				organ regeneration	GO:0031100
				phosphatidylinositol-mediated signaling	GO:0048015
				polysaccharide metabolic process	GO:0005976
				protein homodimerization activity	GO:0042803
				pyrimidine nucleoside biosynthetic process	GO:0046134
				regulation of transcription involved in G1/S transition of mitotic cell cycle	GO:0000083
				small molecule metabolic process	GO:0044281
				tetrahydrofolate metabolic process	GO:0046653
				thymidylate synthase activity	GO:0004799
				uracil metabolic process	GO:0019860
BAK1	BCL2-antagonist/killer 1	578	6p21.31	regulation of apoptotic process	GO:0042981
