# Supplementary material for: Anomalous altered expressions of downstream gene-targets in TP53-miRNA pathways in head and neck cancer
Source: Sci Rep. 2014 Sep 4;4:6280. doi: 10.1038/srep06280 (PMC5385823; doi:10.1038/srep06280)
Supplement: Supplementary Information [file srep06280-s1.docx]

**Anomalous altered expressions of downstream gene-targets in TP53-miRNA pathways in head and neck cancer**

**Sanga Mitra^1^, Nupur Mukherjee^2^, Smarajit Das^3^, Pijush Das^4^,** **Chinmay Kumar Panda^2, #^ & Jayprokas Chakrabarti^1, 5, #^**

^1^Computational Biology Group, Indian Association for the Cultivation of Science, Kolkata, India.

^2^Department of Oncogene Regulation, Chittaranjan National Cancer Research Institute, Kolkata, India.

^3^Department of Medical Biochemistry and Cell Biology, Institute of Biomedicine, University of Gothenburg, Sweden.

^4^Cancer Biology & Inflammatory Disorder Division, Indian Institute of Chemical Biology, Kolkata, India

^5^Gyanxet, BF 286 Salt Lake, Kolkata, India.

^#^Correspondence and requests for materials should be addressed to Chinmay Kumar Panda ([ckpanda.cnci@gmail.com](mailto:ckpanda.cnci@gmail.com)) and Jayprokas Chakrabarti ( [j.chakrabarti@gyanxet.com](mailto:j.chakrabarti@gyanxet.com))

**Supplementary Information**

**Materials and Methods:**

**Expression Data Analysis-**

**Microarray Data.** We downloaded HNSCC microarray data sets from Oncomine version 4.4.4.4. Six datasets were taken under consideration. We followed the supervised learning approach to analyze our data from microarray datasets. Gene-targets of TP53-miRNA pathway were selected for our study. To predict the potential candidates, i.e. those altered in HNSCC, from the 90 gene-targets, the expression value (t-value in our case) were noted from individual datasets. At 95% significant level, the targets lining on either side of the critical value were selected. Based on these values of the targets, collected from all the datasets, Z statistics was applied and support from the z-value was used to rank the 90 genes. Targets were filtered at both 95% confidence interval and 99% confidence interval of z-value (Figure 3a). The targets qualifying 95% confidence interval were chosen for further study. Based on these statistical considerations, it became possible for us to determine the strong oncogenic and tumor suppressive hubs. These selected targets were compared to our HNSCC gene list from HNOCDB. For any test to be performed whether it is t-test or z-test, a certain level of significance was considered, conveniently taken as 5%.

To determine the expression level of miRNA-target genes t-statistics and z-statistics were applied. The normalized fold change data provided for each target from each dataset was considered as background data. t-value was calculated for 90 targets considered and based on t-critical value the filtered targets were selected. Similarly, z-statistics was used as a second sieving criterion. The relevant equations for calculating t-values and z values were:

$$\boldsymbol{t}\boldsymbol{=}\frac{\bar{\boldsymbol{x}\boldsymbol{1}}\boldsymbol{-}\bar{\boldsymbol{x}\boldsymbol{2}}}{\boldsymbol{\sigma}\sqrt{\frac{\boldsymbol{1}}{\boldsymbol{n}\boldsymbol{1}}\boldsymbol{+}\frac{\boldsymbol{1}}{\boldsymbol{n}\boldsymbol{2}}}}$$

$$\boldsymbol{\sigma}\boldsymbol{=}\sqrt{\frac{\boldsymbol{n}\boldsymbol{1}{\boldsymbol{s}\boldsymbol{1}}^{\boldsymbol{2}}\boldsymbol{+}{\boldsymbol{n}\boldsymbol{2}\boldsymbol{s}\boldsymbol{2}}^{\boldsymbol{2}}}{\boldsymbol{n1}\boldsymbol{+}\boldsymbol{n}\boldsymbol{2}\boldsymbol{-}\boldsymbol{2}}}$$

$$\boldsymbol{z}\boldsymbol{=}\frac{\boldsymbol{x}\boldsymbol{-}\bar{\boldsymbol{x}}}{\frac{\boldsymbol{\sigma}}{\sqrt{\boldsymbol{n}}}}$$

$\bar{x1}=Average Expression of a particular gene for a set of Oral cancer patien$t.

$\bar{x2}=Average Expression of a same gene for a set of Control$..

$n1=Number ofpatient$.

$\bar{x2}=Number of Control$.

$s1=Standard deviation of expression value of a particular gene for cancer patient$.

$s2=Standard deviation of expression value of a same gene for control sample$.

In Oncomine median rank score test was used to do the meta-analysis.

**NGS Analysis-**

**RNA-Sequencing Data.** From The Cancer Genome Atlas (TCGA) portal, we downloaded HNSCC data matrix (RNA-Seq data) of Batch 83 consisting of 22 treated samples and 4 untreated samples. The previously explored 90 targets were again used for our NGS study. Our goal was to determine through NGS analysis which of these targets were altered in HNSCC. Based on the read count of the samples, class prediction test, a supervised learning method, was applied. The prediction paradigm served as a good framework for comparing different prediction methods and as a result gave us non-spurious findings. In this analysis 7 different statistical methods, namely “Compound Covariate Predictor”, “Diagonal Linear Discriminant Analysis”, “1-Nearest Neighbor”, “3-Nearest Neighbors”, “Nearest Centroid”, “Support Vector Machines” and “Bayesian Compound Covariate Predictor” were introduced to classify each individual samples (case/control) with the basis of classifier genes. Prediction strengths were evaluated for all genes on the considered gene list using data from the given samples. Genes with highest predictive strength of the cancer and normal classes were selected equally to generate a final list of best predictor genes.

**Class Prediction Test.** The prediction paradigm will serve as a good framework for comparing different prediction methods and may accelerate the development of molecular classifiers that are clinically useful. The read count data of HNSCC samples were normalized using Lima package. Next, we proceeded for class prediction operation. Class prediction operation had been performed with multiple methods: 1) Compound covariate predictor 2) Bayesian Compound Covariate 3) Diagonal linear discriminant analysis 4) K-nearest neighbor (for k=1, and k=3) 5) Nearest centroid and 6) Support vector machines. Leave one out cross-validation method was implemented and the number of permutation for significance test of cross-validated mis-classification rate had been taken 100.

**Negative binomial Test****.** RNA sequencing (RNA-Seq) based gene expression with much improved accuracy and sensitivity identified a set of transcripts that were differentially expressed between different experimental conditions. Recently several methods were developed specifically for RNA-Seq data sets to detect the differentially expressed genes from a read count. As the number of samples or replicates in a typical RNA-Seq experiment were usually small; therefore, we focused on just the parametric methods. These methods differed in their underlying data distributions, how they handled biological replicates, and their ability to perform multi-group comparisons. Three major statistical methods emerged for RNA-Seq differential gene expression analysis under the distribution of Poisson, Negative Binomial and Beta Binomial. Now every distribution had its own advantage, but when over-dispersion was observed across the samples, the gene counts could be estimated accurately by a simple Poisson model. One way to handle this problem was to apply a Bayesian method – allowing the Poisson mean follows a Gamma distribution. We applied Negative Binomial distribution that can model the over-dispersed Poisson gene count and reduces to the Poisson distribution. The R/Bioconductor package “DeSeq” applied this model to detect the differentially expressed genes in RNA-Seq data^1^.

NB distribution generated four differentially expressed values of mRNA data that are fold change, t value, p value and q value. We chose q value for further discrimination of data. A certain level of significance was considered to filter our data, conveniently taken as 5%. To minimize this error Bonferroni correction or a modified one by Holm’s may be applied on p-value but both these methods lead to over killing of false positive. Reducing stringency of above two methods Benjamini and Hochhberg designed False Discovery Rate (FDR) to control the proportion of false positives among the set of rejected hypotheses. q value is the FDR analogue of p-value.

**Expression Analysis by qRT-PCR.** The expression of the candidate genes (MEIS1, LDOC1, AGTR1, BAK1, TYMS and DTL) in HNSCC primary tumor (n=1) and 3 cell lines (Hep2, KB and SCC 084) were analyzed by quantitative Real-Time PCR and then compared its expression with the normal head and neck tissues (n=3), using SYBR-green PCR assay and β2-Microglobulin gene as control. Primer pairs used for expression analysis are given in Table S1.

Total RNA was isolated from samples using TRIzol reagent according to the manufacturer’s protocol (Invitrogen). Reverse transcription was performed with 1 µg total RNA using Random hexamer (Invitrogen) and Superscript III (Invitrogen). Briefly, 2 µl of cDNA was amplified in a 25 µl reaction solution containing 12.5 µl 2XSYBR-green master mix (Applied Biosystems, Foster City, CA) and 1 µl of each primer. Each sample was loaded in triplicate and run at 40 cycles on an ABI prism 7500 machine (Applied Biosystems). After each run, melting curves were generated to confirm amplification of specific transcripts.

To determine the relative level of gene expression, the comparative threshold cycle (ddC_T_) method was employed after normalization of respective gene of interest expression level against that of *B2M*, (dC_T_)^2^. The relative gene expression of the candidate genes were calculated from the *C*T values obtained using the 2-ΔΔC_T_ Method where:

**References**

1. Anders, S. & Huber, W. Differential expression analysis for sequence count data. *Genome Biol.* **11,** R106 (2010)
2. Livak, K.J. & Schmittgen, T.D. Analysis of relative gene expression data using real-time quantitative PCR and the 2(-Delta Delta C(T)) Method. *Methods* **25,** 402–408 (2001).

**Table S1:** Oligonucleotide primers used for mRNA expression analysis in the study

| **Primers** | **Analytical purpose** | **Forward Primer** | **Reverse Primer** | **Size (bp)** |
| --- | --- | --- | --- | --- |
| MEIS1 Exon4-6 | mRNA expression | 5'-TCGCGCAGAAAAACCTCTAT -3' | 5'- GGCATTTTCCCTTTCAAAC -3' | 162 |
| LDOC1 Exon1 | mRNA expression | 5'-CCTTCCCCGAAACGTTTAAT -3' | 5'-AGGATGGGGCTATCCATCT -3' | 190 |
| AGTR1Exon4 | mRNA expression | 5'-CCTGCATCATCATTTGG-3' | 5'-GGGTTGAATTTTGGGACTCA -3' | 129 |
| BAK1 Exon5-6 | mRNA expression | 5'-TCTGGCCCTACACGTCTACC -3' | 5'-ACAAACTGGCCCAACAGAAC -3' | 201 |
| TYMS Exon2-3 | mRNA expression | 5'-TCTGGAAGGGTGTTTTGGAG -3' | 5'-CCTCCACTGGAAGCCATAAA -3' | 182 |
| DTL Exon3-5 | mRNA expression | 5'-CTTCAAAGAATGGATGGCTCA -3' | 5'-TTTGGCTGTTTGATCACCTG -3' | 100 |
| β2 Microglobulin | Control for expression | 5'-GTGCTCGCGCTACTCTCTCT-3' | 5'-TCAATGTCGGATGGATGAAA-3' | 153 |
